# Pott’s Puffy Tumor: A Case Report on Diagnosis Through Imaging

**DOI:** 10.7759/cureus.58640

**Published:** 2024-04-20

**Authors:** Jonathan Pimiento Figueroa, Marcia Mejia, Daniela Bohorquez

**Affiliations:** 1 Radiology, Servicios de Salud San Vicente Fundación, Medellín, COL; 2 Radiology, Universidad de Antioquia, Medellín, COL; 3 Otolaryngology, Universidad de Antioquia, Medellín, COL

**Keywords:** periorbital cellulitis, craniofacial osteomyelitis, intracranial epidural abscess, acute sinusitis, pott's puffy tumor

## Abstract

Pott's puffy tumor (PPT) is a rare but life-threatening complication of chronic sinusitis, although it can be secondary to other entities such as trauma or insect bites. It is characterized by circumscribed frontal swelling associated with a subperiosteal abscess. Imaging plays a crucial role in diagnosis and early identification of complications, some of which can be life-threatening, including intracerebral and intra-orbital complications. We present a case of a 14-year-old male with non-specific frontal pain and swelling, where the diagnosis of PTT was confirmed through imaging studies. Upon admission, the patient exhibited orbital and intracerebral complications, as shown in MRI and CT scans. Treatment involved a combination of antibiotics and sinus surgery, with close monitoring for orbital and intracranial complications.

## Introduction

Pott's puffy tumor (PPT) is characterized by localized swelling on the forehead linked to edema and a frontal subperiosteal abscess [1,2]. It typically stems from untreated or undiagnosed sinusitis, though it can also be connected to other factors such as trauma or insect bites [3]. While it is deemed a rare condition, the potential complications can pose serious risks, such as intracranial and intraorbital issues [3].

Diagnostic images play a crucial role in the diagnostic process by aiding in confirming the diagnosis and ruling out complications that may necessitate further treatment [1]. These complications encompass a range from orbital to intracranial issues, such as orbital cellulitis, endophthalmitis, intraorbital abscess, epidural abscess, subdural abscess, and dural sinus thrombosis [4].

Treatment relies on promptly starting antibiotics and performing timely surgical interventions for local infection control. This includes endoscopic sinunasal surgery, draining subperiosteal abscesses, and potentially addressing orbital and intracranial abscesses based on associated complications [5]. The prognosis, typically positive, hinges greatly on timely diagnosis and treatment initiation [1].

## Case presentation

A 14-year-old male patient with no significant medical history presented to the ER with a three-day history of frontal pain, accompanied by nausea, which worsened with physical activity. He denied having fever, visual, auditory, or respiratory symptoms. Physical examination revealed edema in the frontal region, tender to touch, without redness, warmth, or skin lesions. At the ear, nose, and throat examination, postnasal drip was detected. No significant abnormalities were noted in the neurological or ear examinations. Initially suspected of neuralgia, he was treated with non-steroidal anti-inflammatory drugs (NSAIDs), steroids, and metoclopramide, resulting in partial pain relief. Neurologists evaluated the patient, found no additional alterations in the physical examination, and deemed the etiology unclear. They considered complementing the study with a simple CT scan of the skull and paranasal sinuses.

**Figure 1:**
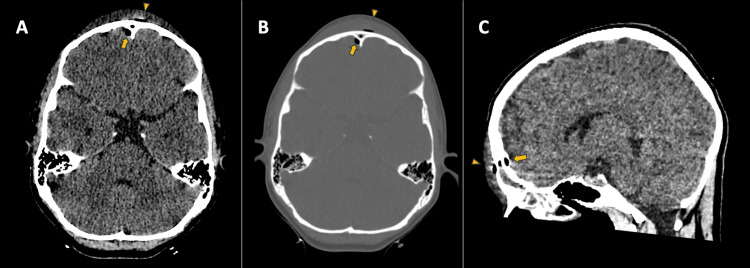
Simple skull tomography. A. Axial - Brain Window, B. Axial - Bone Window, C. Sagittal - Brain Window. Frontal pneumoencephalus (Arrow). Frontal soft tissue edema with an air bubble near the anterior wall of the frontal sinus (Arrowhead).

**Figure 2:**
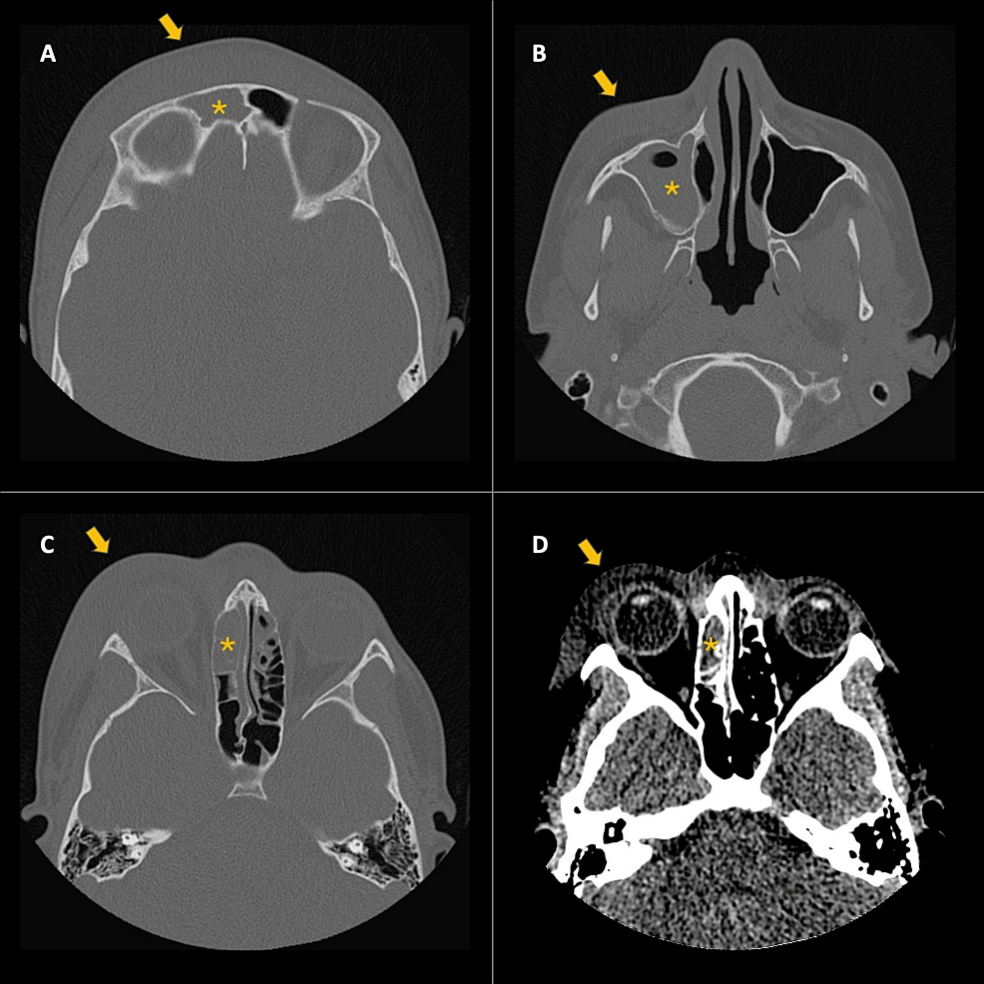
Simple tomography of the paranasal sinuses. A. Axial - Bone Window: Thickening and edema of the frontal soft tissues (Arrow). Right frontal sinus occupied (Asterisk). B. Axial - Bone Window: Thickening and edema of the malar soft tissues on the right side (Arrow). Right maxillary sinus occupied with an air-fluid level (Asterisk). C-D. Axial - Bone and Soft Tissue Window: Thickening of the right periorbital, preseptal soft tissues (Arrow). Right ethmoidal cells occupied (Asterisk).

An initial head CT scan revealed minimal right frontal pneumoencephalus, inflammatory changes in the soft tissues of the frontal region and upper eyelid, and subcutaneous emphysema near the anterior wall of the frontal sinus (Figure 1). Similarly, a CT scan of the paranasal sinuses showed opacification of the frontal sinus, ethmoid cells, and right maxillary sinus with a fluid/liquid level in the latter, as well as blockage of the ipsilateral osteomeatal complex (Figure 2). Based on these results, a diagnosis of complicated sinusitis associated with PPT was established. Due to the presence of pneumoencephalus in the CT scans, a contrast-enhanced MRI of the skull was requested to rule out a brain abscess. Antibiotic treatment was initiated with vancomycin and ceftriaxone.

**Figure 3:**
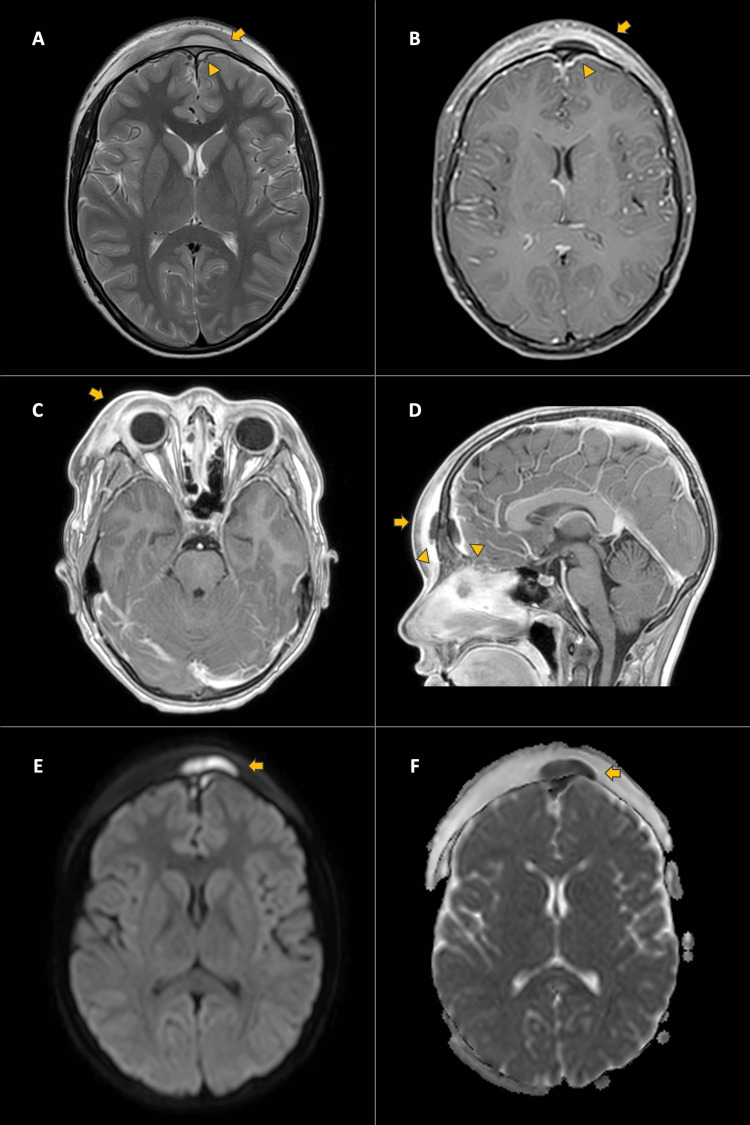
Contrast-enhanced MRI of the skull. A. T2 - Axial: Subperiosteal collection, observe the periosteum lifting depicted as a thin hypointense line (arrow). Bilateral frontal epidural collection (arrowhead). B. Contrast-enhanced SPIR - Axial: Frontal soft tissue enhancement surrounding the frontal collection (arrow). Leptomeningeal enhancement adjacent to frontal epidural collection (arrowhead). C. Contrast-enhanced T1 - Axial: Enhancement of the pre- and post-septal periorbital soft tissues (arrow). D. Contrast-enhanced T1 - Sagittal: Enhancement of the soft tissues of the forehead (arrow). Note the collection in the frontal soft tissues and frontal epidural, the latter with adjacent leptomeningeal enhancement (arrowheads). E-F. DWI and ADC Map: Frontal collection (arrows) hyperintense in the diffusion sequence and hypointense in the ADC map; restriction in the diffusion sequences confirms the presence of pus. T2: T2-weighted MRI; T1: T1-weighted MRI; DWI: Diffusion-weighted imaging; ADC: Apparent diffusion coefficient; SPIR: Spectral presaturation with inversion recovery.

In the contrast-enhanced MRI of the brain, inflammatory changes are observed in the pre- and post-septal soft tissues of the right orbit, right frontal epidural collection, and subperiosteal collection in the anterior wall of the frontal sinus, in addition to the findings noted in tomographic studies (Figure 3).

Based on the imaging results, the ophthalmology team considers non-surgical management for right orbital cellulitis; neurosurgery opts for non-surgical management of the frontal epidural abscess; and otorhinolaryngology decided to perform transnasal endoscopic surgery. The examination revealed a blockage of the osteomeatal complex due to inflammatory tissue and a small amount of pus in the right maxillary sinus and frontal recess on that side. Additionally, an external supraciliary approach was taken, uncovering a subperiosteal collection holding around 8cc of pus.

**Figure 4:**
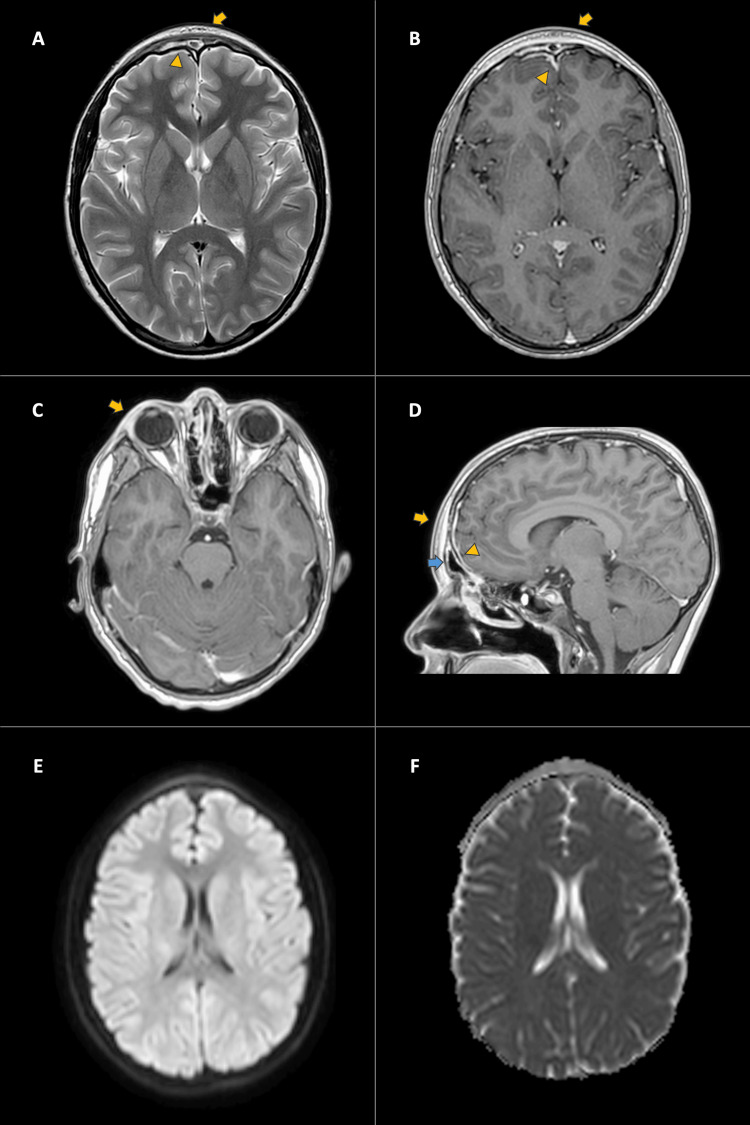
Contrast-enhanced MRI of the skull (control). A. T2 - Axial: Resolution of inflammatory changes and abscess in the frontal soft tissues (Arrow). Residual thickening and enhancement of the frontal sinus (Arrowhead). B. Contrast-enhanced T1 - Axial: Resolution of inflammatory changes and collection in the frontal soft tissues (Arrow). Residual leptomeningeal enhancement (Arrowhead). Resolution of frontal epidural collection. C. Contrast-enhanced T1 - Axial: Significant reduction in edema and enhancement of the right periorbital soft tissues (Arrow). D. Contrast-enhanced T1 - Sagittal: Resolution of inflammatory changes and collection in the soft tissues of the forehead (Yellow Arrow). Residual thickening and enhancement of the frontal sinus (Blue Arrow). Residual leptomeningeal enhancement (Arrowhead). E-F. DWI and ADC Map: No areas of restricted diffusion due to the resolution of epidural and frontal soft tissue abscesses. T2: T2-weighted MRI; T1: T1-weighted MRI; DWI: Diffusion-weighted imaging; ADC: Apparent diffusion coefficient.

Following this, Staphylococcus aureus susceptible to oxacillin was found in the purulent fluid, leading to a modification in antibiotic therapy from vancomycin/ceftriaxone to oxacillin. The patient's progress was positive, as evidenced by a follow-up MRI conducted 19 days after the initiation of antibiotic treatment (Figure 4). Additionally, trimethoprim-sulfamethoxazole was included in the treatment regimen based on the recommendations of an infectious disease specialist, resulting in satisfactory clinical improvement during subsequent medical evaluations. The patient was discharged without any apparent sequelae. At the follow-up visit after discharge, the patient was asymptomatic.

## Discussion

PPT is a localized swelling on the forehead caused by osteomyelitis of the anterior wall of the frontal sinus and a subperiosteal abscess, leading to edema of the underlying skin [1,2]. Sir Percival Pott first described this condition in the 18th century, linking it to complications from frontal trauma [6]. Nowadays, it is commonly associated with a history of untreated or poorly managed frontal sinusitis; however, it can also be triggered by factors such as trauma in individuals with sinusitis, previous frontal surgery, cocaine use, insect bites, fibrous dysplasia, or dental infections [3].

PPT has become less common in the post-antibiotic era, with reports in the medical literature being rare; however, it is believed to be more prevalent than is suggested in the medical literature [3]. This condition is influenced by the structure of the frontal sinuses and the diploic veins that drain them, which aid in the spread of infection from the sinuses to the bone and brain. While PPT can affect individuals of all ages, it is more frequent in adolescents. This increased prevalence is attributed to the flow of diploic veins draining the mucosa of the frontal sinuses and promoting the spread of infection through the bloodstream, coinciding with the maturation of the frontal sinuses [1,3].

Symptoms associated with PPT include swelling on the forehead that can extend to the orbit, frontal headache, purulent or clear rhinorrhea, and fever [5]. Other symptoms may include nausea, vomiting, cutaneous fistulas, as well as neurological and visual symptoms, depending on the presence or absence of intracerebral and ocular complications, respectively [7]. Laboratory tests are typically useful, showing an elevation in acute phase reactants and leukocytes [5].

While the diagnosis of PPT can be determined from the clinical history and physical examination findings, diagnostic images are crucial in the diagnostic process, with at least one brain image being required [8-10]. The selection of an imaging method considers various factors such as availability, cost, and the technical benefits of each modality, including improved contrast resolution and the lack of ionizing radiation in MRI, quicker acquisition times, and the widespread availability of CT [11].

Imaging presentations in medical literature for diagnosing PPT primarily focus on CT and MRI studies. Some cases have shown ultrasound to be particularly helpful in confirming the diagnosis [9,12]. CT and MRI results are typically similar and serve as complements to each other, often requiring the use of both techniques [4]. Typical findings include paranasal sinus occupation, erosion of the frontal sinus bony walls, periorbital inflammation, pneumoencephalus, subperiosteal collection, leptomeningeal enhancement, epidural collection, and dural sinus thrombosis [13].

Contrast-enhanced CT is often the initial choice for assessing PPT due to its speed and accessibility. It is also considered superior in detecting bone erosions [4,14]. Conversely, MRI is the preferred method for identifying intracranial changes and is essential when such alterations are suspected [1]. Moreover, it enables the characterization of intra- and extracranial accumulations through diffusion sequences [4].

The management of PPT often necessitates an interdisciplinary team involving specialists in infectious diseases, otorhinolaryngology, neurosurgery, and ophthalmology, based on the associated complications during hospitalization and follow-up [4]. The prognosis of PPT relies on swift diagnosis and treatment; hence, treatment should commence promptly to avert and address life-threatening complications linked to PPT [7]. Key treatment components include timely antibiotic administration and the prompt execution of surgical interventions for managing the local infectious process, including endoscopic sinunasal surgery, subperiosteal abscess drainage, and, if indicated, drainage of intracranial or intraorbital abscesses [1].

## Conclusions

PPT presents with circumscribed frontal swelling and a subperiosteal abscess in the anterior wall of the frontal sinus. As in our case, sinonasal infections, whether silent, undiagnosed, or poorly treated, are the main cause. While clinical history and examination usually lead to a diagnosis, imaging is essential for confirmation and for assessing the extent of the infection and potential complications. Treatment involves the early administration of antibiotics and local infection control through a multidisciplinary approach, including specialists in otorhinolaryngology, neurosurgery, ophthalmology, and infectious diseases. The prognosis is generally satisfactory with timely diagnosis and treatment.
